# Selective and Efficient Elimination of *Vibrio cholerae* with a Chemical Modulator that Targets Glucose Metabolism

**DOI:** 10.3389/fcimb.2016.00156

**Published:** 2016-11-16

**Authors:** Young Taek Oh, Hwa Young Kim, Eun Jin Kim, Junhyeok Go, Wontae Hwang, Hyoung Rae Kim, Dong Wook Kim, Sang Sun Yoon

**Affiliations:** ^1^Department of Microbiology and Immunology, Yonsei University College of MedicineSeoul, South Korea; ^2^Brain Korea 21 Project for Medical Science, Yonsei University College of MedicineSeoul, South Korea; ^3^Department of Pharmacy, College of Pharmacy, Hanyang UniversityAnsan, South Korea; ^4^Bio and Drug Discovery Division, Korea Research Institute of Chemical TechnologyDaejeon, South Korea; ^5^Institute of Pharmacological Research, Hanyang UniversityAnsan, South Korea; ^6^Institute for Immunology and Immunological Diseases, Yonsei University College of MedicineSeoul, South Korea

**Keywords:** *Vibrio cholerae*, glucose metabolism, acetoin, mixed acid fermentation, inducer of medium acidification (iMAC)

## Abstract

*Vibrio cholerae*, a Gram-negative bacterium, is the causative agent of pandemic cholera. Previous studies have shown that the survival of the *seventh* pandemic El Tor biotype *V. cholerae* strain N16961 requires production of acetoin in a glucose-rich environment. The production of acetoin, a neutral fermentation end-product, allows *V. cholerae* to metabolize glucose without a pH drop, which is mediated by the production of organic acid. This finding suggests that inhibition of acetoin fermentation can result in *V. cholerae* elimination by causing a pH imbalance under glucose-rich conditions. Here, we developed a simple high-throughput screening method and identified an inducer of medium acidification (iMAC). Of 8364 compounds screened, we identified one chemical, 5-(4-chloro-2-nitrobenzoyl)-6-hydroxy-1,3-dimethylpyrimidine-2,4(1H,3H)-dione, that successfully killed glucose-metabolizing N16961 by inducing acidic stress. When N16961 was grown with abundant glucose in the presence of iMAC, acetoin production was completely suppressed and concomitant accumulation of lactate and acetate was observed. Using a beta-galactosidase activity assay with a single-copy p*alsD::lacZ* reporter fusion, we show that that iMAC likely inhibits acetoin production at the transcriptional level. Thin-layer chromatography revealed that iMAC causes a significantly reduced accumulation of intracellular (p)ppGpp, a bacterial stringent response alarmone known to positively regulate acetoin production. *In vivo* bacterial colonization and fluid accumulation were also markedly decreased after iMAC treatment. Finally, we demonstrate iMAC-induced bacterial killing for 22 different *V. cholerae* strains belonging to diverse serotypes. Together, our results suggest that iMAC, acting as a metabolic modulator, has strong potential as a novel antibacterial agent for treatment against cholera.

## Introduction

Acetoin, known as 3-hydroxy-2-butanone or acetyl methyl carbinol, is produced by various microorganisms in which acetoin often has physiological functions required for survival (Voloch et al., 1983; Yoon and Mekalanos, 2006; Vivijs et al., 2014a,b). In *Vibrio cholerae*, enzymes, encoded by *VC1589-VC1590* locus are involved in acetoin biosynthesis. The α-acetolactate synthase (*VC1590*, AlsS) catalyzes the conversion of two molecules of pyruvate into α-acetolactate, and α-acetolactate decarboxylase (*VC1589*, AlsD) subsequently catalyzes the decarboxylation of α-acetolactate into acetoin (Kovacikova et al., 2005; Xiao and Lu, 2014). Of additional note, *V. cholerae* N16961 produces a LysR-type transcriptional regulator (referred to as AlsR). Expression of *alsR* gene is induced by environmental factors such as acetate, anaerobiosis, or low environmental pH, and it activates the expression of the acetoin biosynthesis genes (Renna et al., 1993; Mayer et al., 1995; Frädrich et al., 2012). Because acetoin is a neutral fermentation end product and this biosynthetic reaction consumes intracellular protons, bacterial growth can occur on a glucose carbon source without pH decrease (Thomas et al., 2014). These findings indicate that acetoin production during fermentation plays an important physiological role in the adaptation to acidic stress, and it also suggests a potential mechanism to control pathogenic bacteria like *V. cholerae*, that are known to be acid-sensitive (Merrell and Camilli, 2002).

The Gram-negative bacterium *V. cholerae* is the etiological agent of cholera, a life-threatening severe diarrheal disease (Faruque et al., 1998; Sack et al., 2004). The pathogen produces two major virulence factors: the cholera toxin (CT), which is responsible for massive watery diarrhea by causing an imbalance of ion transport across the intestinal epithelium; and the toxin-coregulated pilus (TCP), which contributes to the successful colonization of *V. cholerae* in the host intestine (Miller et al., 1987; Herrington et al., 1988; Thelin and Taylor, 1996; Childers and Klose, 2007). Previous work has demonstrated that the seventh pandemic *V. cholerae* O1 El Tor biotype strains produce acetoin, a metabolite that provides a growth advantage under glucose-rich conditions over the classical biotype strains (Yoon and Mekalanos, 2006). Further, it is proposed that the production of acetoin may account for the greater evolutionary fitness of *V. cholerae* El Tor biotype compared to the classical biotype (Yoon and Mekalanos, 2006). Additionally, acetoin production offers *V. cholerae* cells a survival advantage during infection by down regulating the host innate immune responses (Bari et al., 2011). These findings support the hypothesis that inhibiting acetoin production could provide a novel antibacterial mechanism for *V. cholerae* elimination.

In *V. cholerae*, there are two major regulatory mechanisms for acetoin biosynthesis. First, acetoin production is negatively regulated by AphA, a quorum sensing (QS)-dependent transcriptional activator. AphA represses expression of the acetoin biosynthetic genes by binding to a region that overlaps the transcriptional start site of *alsD* (Kovacikova et al., 2005). Meanwhile, it also reduces the expression of the divergently transcribed *alsR* gene, which encodes an acetate-responsive LysR-type regulator (Kovacikova et al., 2005). Second, recent work from our group has shown that (p)ppGpp, a stringent response alarmone, also plays a critical role in regulating acetoin production. A mutant strain defective in (p)ppGpp production lost the ability to produce acetoin and becomes inviable due to uncontrolled production of organic acids in the presence of glucose (Oh et al., 2015). We also demonstrated that, in the seventh pandemic N16961 El Tor strain, (p)ppGpp-mediated regulation is more important for maintaining bacterial viability than the QS-dependent regulation, which is controlled in an AphA-dependent manner (Oh et al., 2015). These findings strongly suggest that (p)ppGpp is a promising drug target for the inhibition of *V. cholerae* infection and survival.

In this study, we screened a chemical library and identified a compound that induces medium acidification and thereby loss of *V. cholerae* viability under glucose-rich conditions. Further, we present a potential mechanism by which the compound inhibits acetoin biosynthesis. By targeting acetoin biosynthesis, our work provides a novel strategy to combat cholera.

## Materials and methods

### Ethics statement

All animal experiments were conducted following national guidelines provided by the Korean government (Ministry for Food, Agriculture, Forestry, and Fisheries) and in strict accordance with the institutional guidelines for animal care and use of laboratory animals. The methods for animal experimentations using infant mouse were approved by the Committee on the Ethics of Animal Experiments of the Yonsei University College of Medicine (Permit Number 2011-0166).

### Bacterial strains and growth conditions

All bacteria strains used in this study are listed in Table S1. *V. cholerae* strains and *E. coli* DH5α strain cultures were grown at 37°C in LB medium (LB; 1% (w/v) tryptone, 0.5% (w/v) yeast extract, and 1% (w/v) NaCl). *Vibrio vulnificus* MO6-24/O strain and *Vibrio paraheamolyticus* ATCC27519 strain were grown at 30°C in LBS [LB medium containing NaCl at a final concentration of 2.5% (w/v)] media. A single colony grown on an LB agar plate was picked and inoculated to start a pre-culture. Overnight cultures were then diluted 100-fold in LB or LBG [LB containing 1% glucose (w/v)] for the main cultures. When necessary, 200 μg/ml streptomycin was used for selective *V. cholerae* growth. Bacterial growth was measured spectrophotometrically according to optical density at 600 nm. The pH of the culture media was measured using a pH meter (METTLER TOLEDO Co. pH S20-K MASA). To describe cell viability, the number of colony forming units (CFUs) was quantified by plating serial dilutions of bacterial cultures.

### A high-throughput screening assay to identify inducers of metabolism-mediated acidification (iMAC)

To identify iMAC candidates, a 8364-compound library, obtained from the Korea Chemical Bank (Daejoen, Korea), was used in our screening assay. Overnight-grown bacterial cells were diluted 100-fold in LB or LBG for primary screening cultures, and 200 ul of each were inoculated on a 96-well plate containing glass beads for effective aeration. Each chemical in the screen was added in LB and LBG at 50 μM final concentration, and cells were grown in aerobic conditions for 16 h with shaking. To verify the pH of each culture supernatant, phenol red (20 mg/liter; Sigma Chemical Co., St. Louis, MO) was added to bacterial cell cultures as a pH indicator. One compound of chemical library, 5-(4-chloro-2-nitrobenzoyl)-6-hydroxy-1,3-dimethylpyrimidine-2,4(1H,3H)-dione (iMAC), (**Figure 2A**) was selected for further study, and it was subsequently synthesized in a large amount from the Korea Research Institute of Chemical Technology (KRICT, Daejoen, Korea). Initial iMAC stock solution was made at 5 mM in 10% DMSO (dimethyl sulfoxide). Then, the stock solution was diluted in LB or LBG.

### Quantification of metabolites

To quantify the amounts of organic acids in the culture media, bacterial cells were grown aerobically for 16 h in LB, LBG, or LBG + 50 μM iMAC media and subsequently harvested by centrifugation at 14,000 rpm for 10 min. The culture supernatants were filtered using 0.22 μm-pore syringe filters (Pall Corp., MI, USA), and samples were analyzed using high-performance liquid chromatography (HPLC). Acetoin level was quantitatively measured using the *Voges-Proskauer* (VP) test as described previously (Kovacikova et al., 2005). Briefly, 5% α-naphthol and 40% KOH were added to cell-free culture supernatant, the reaction mixture was incubated for 30 min at room temperature, and then the value of acetoin amount was determined by measuring absorbance at 490 nm with a spectrophotometer.

### Scanning electron microscopy (SEM)

Overnight cultures grown in LB or LBG media with or without 50 μM iMAC were visualized by scanning electron microscopy (SEM) in order to visualize cell morphology. For the SEM sample preparation, cells were fixed with PBS containing 2% glutaraldehyde and 0.1% paraformaldehyde for 2 h. Samples were then coated with gold by an ion sputter (IB-3 Eiko, Japan) and examined with a scanning electron microscope (FE SEM S-800, Hitachi, Japan) at an acceleration voltage of 20 kV. Images were processed with ESCAN 4000 software (Bummi Universe Co., LTD, Seoul, Korea).

### Thin layer chromatography

Intracellular small nucleotide levels were measured as previously described with slight modification (He et al., 2012). To detect ppGpp concentrations, overnight-grown cultures were diluted 100-fold in LB or LB + 50 μM iMAC media supplemented with 100 μCi/ml of [^32^P]-orthophosphate (Perkin-Elmer, Waltham, MA) and grown at 37°C with shaking for 4 h. The cultures were then extracted with 19 M formic acid. Briefly, bacterial cells were precipitated by centrifugation to remove the cell supernatant, after which the cell pellets were resuspended in cold 10 mM Tris-HCl buffer (pH 8.0) and 19 M formic acid and then subjected to three freeze-thaw cycles. After further centrifugation to remove cell debris, the supernatants were spotted on a polyethyleneimine (PEI)-coated thin-layer chromatography (TLC) plate (Merck & Co., Whitehouse Station, NJ). The plate was developed in 1.5 M KH_2_PO_4_ (pH 3.5) and visualized by autoradiography.

### CT-ELISA and β-galactosidase activity assay

CT production was quantified by subjecting *V. cholerae* culture supernatants to a GM_1_-enzyme-linked immunosorbent assay (GM_1_-ELISA), as described previously (Gardel and Mekalanos, 1994). To collect the culture supernatant, wild-type N16961 strain growth was induced in both CT-inducible culture methods: anaerobic TMAO respiration condition and the AKI condition, as described previously (Iwanaga et al., 1986). Bacterial cells were subsequently centrifuged at 13,000 rpm and 4°C for 20 min. Cell-free supernatants were then collected and passed through 0.22 μm-pore syringe filters (Pall Corp., MI, USA). Purified CT subunit B (List Biological Laboratories, Inc., Campbell, CA) was used to provide a standard curve, and phosphate-buffered saline (PBS) was used as a negative control. Rabbit polyclonal anti-CT subunit B (Abcam Inc., Cambridge, UK) and anti-rabbit immunoglobulin G conjugated with horseradish peroxidase (Santa-Cruz Biotechnology Inc., Dallas, TX) were used to detect the presence of CT. The promoter activity of *alsD* (*VC1598*) was assessed by measuring β-galactosidase activity in the wild-type N16961 strain containing a p*alsD::lacZ* fusion gene constructed previously (Oh et al., 2015). Bacterial cells were harvested after incubation for 5 h in LB, LBG, or LBG + 50 μM iMAC, and β-galactosidase activity was assayed as described previously (Yoon and Mekalanos, 2006).

### Infant mouse infection

Bacterial strains were suspended in LB or LBG with or without 50 μM iMAC to a final cell density of 1 × 10^7^ CFU/mL. Infant mice (~5–6 days old, Central Lab Animal Inc., Seoul) were orogastrically infected with 50 μL of each bacterial suspension (5 × 10^5^ cells). After 24 h of infection, the entire intestine was removed and homogenized in 2 mL PBS. Bacterial cell viability was determined by enumerating colony numbers of serially diluted gut homogenates. LB agar plates supplemented with streptomycin (100 μg/ml) were used for CFU counting. The fluid accumulation ratio is calculated as the gut weight/remaining body weight.

### Cytotoxicity assay

Host cell culture was performed as described previously (Bari et al., 2011). Intestinal epithelial HT29 cells (ATCC, Rockville, MD, USA) were cultured in Dulbecco's Modified Eagle Medium (DMEM) supplemented with 10% FBS (WelGENE, Korea), 2.5 mM L-glutamine, 100 U/mL penicillin, and 100 μg/mL streptomycin at 37°C in a 5% CO_2_ atmosphere in a water-jacketed incubator (Forma Scientific, USA). Then, 2 × 10^5^ HT29 cells were seeded per well in 6-well plates (SPL Life Sciences, Korea) and cultured overnight. The following day, culture media was replaced with serum-free media prior to treatment. Various concentrations of iMAC were then added to the host cells, and mixtures were incubated for 48 h at 37°C under 5% CO_2_. Images of viable cells were visualized by optical microscopy. The cytotoxicity assay was performed using colorimetric assay as described previously (Mosmann, 1983). Samples of culture media were then collected for the cell viability assay using 3-(4, 5-dimethylthiazol-2-yl)-2,5-diphenyltetrazolium bromide tetrazolium reduction assay (MTT assay; Thiazolyl Blue Tetrazolium Bromide, Sigma) according to the manufacturer's instructions.

### Statistical analysis

Data are expressed as mean ± *SD*. Unpaired Student's *t*-test and the Wilcoxon Rank-Sum test (for animal experiments) were used to analyze the data. A *p* < 0.05 (Student's *t*-test) or <0.01 (Wilcoxon test) was considered statistically significant.

## Results

### Identification of a novel chemical compound that induces acidification of the media during glucose metabolism

**Figure 1:**
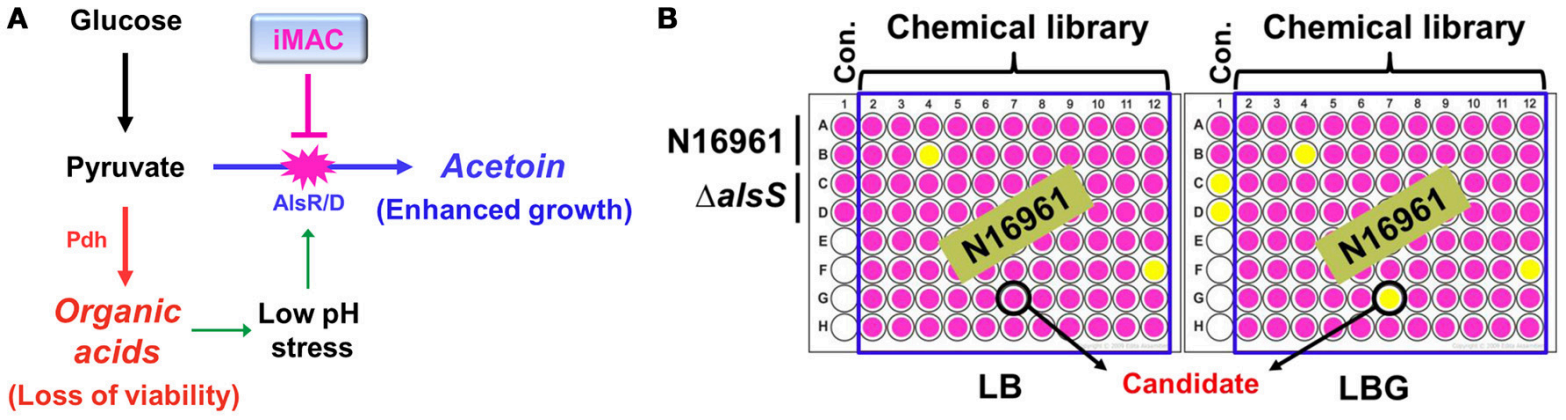
**Schematic diagram of the experimental procedures to identify chemical compounds that inhibit acetoin fermentation. (A)** The strategy of *V. cholerae* elimination using a chemical inhibitor that is capable of suppressing acetoin fermentation. **(B)** A high- throughput screening assay used to identify iMAC. Bacterial cells were inoculated in LB or LBG (LB containing 1% glucose) on a 96-well plate containing glass beads for effective aeration and were grown in aerobic conditions for 16 h. To verify the pH of culture supernatants, phenol-red was used as a pH indicator.

*V. cholerae* O1 El Tor strains grow extremely well in a glucose-rich environment (Yoon and Mekalanos, 2006). The uninterrupted synthesis of acetoin, a neutral fermentation end-product, is a critical component of this robust growth. An N16961 Δ*alsS* mutant that failed to produce acetoin lost its viability for glucose-stimulated growth due to medium acidification. Therefore, we expect significant therapeutic potential if we can prevent *V. cholerae* cells from producing acetoin in glucose-rich media (summarized in Figure 1A). To this end, we designed a chemical library screening method to search for compounds that induce acidification during glucose-induced growth. An initial screen was performed with 8364 chemical compounds obtained from the Korea Chemical Bank. N16961 and its Δ*alsS* mutant were grown in LB or LB plus 1% glucose (LBG) with each chemical compound. Bacterial growth was monitored by measuring OD_600_, and the pH of the media was measured with a pH indicator. Schematic screening procedures are depicted in Figure 1B. From our initial screen, we identified 24 chemicals with the ability to induce medium acidification during N16961 growth in LBG (Figure S1A). In each case, defective growth was concomitantly observed (Figure S1B). Compounds were applied at a concentration of 50 μM. Bacterial growth was not noticeably affected by these compounds in LB, suggesting that the inhibitory effect of each of these chemicals requires the addition of glucose. When viable cells were enumerated in each acidified medium, a reduction in viability was invariably observed (Figure S1C). The number of viable cells of N16961 was ~10-fold higher after growth in LBG vs. LB (Figure S1C), further confirming a glucose-induced effect on the active growth of N16961.

We next sought to determine which compound most effectively inhibits glucose-stimulated growth of N16961. To address this issue, N16961 cells were grown with varying concentrations (50, 25, and 12.5 μM) of each of the 24 positively identified compounds in LBG. Several compounds (numbered as 242411, 198039, 243142, 240997, 207967, 246452, 243275, and 246475) induced acidification of the media at 25 μM, but failed to show a significant effect at 12.5 μM. One compound (17191), however, induced acidification at a 12.5 μM concentration (Figure S2). We therefore selected compound 17191 for further study (herein referred to as iMAC, inducer of Medium ACidification).

### iMAC induces *V. cholerae* cell death in a glucose-rich environment, which normally stimulates growth

**Figure 2:**
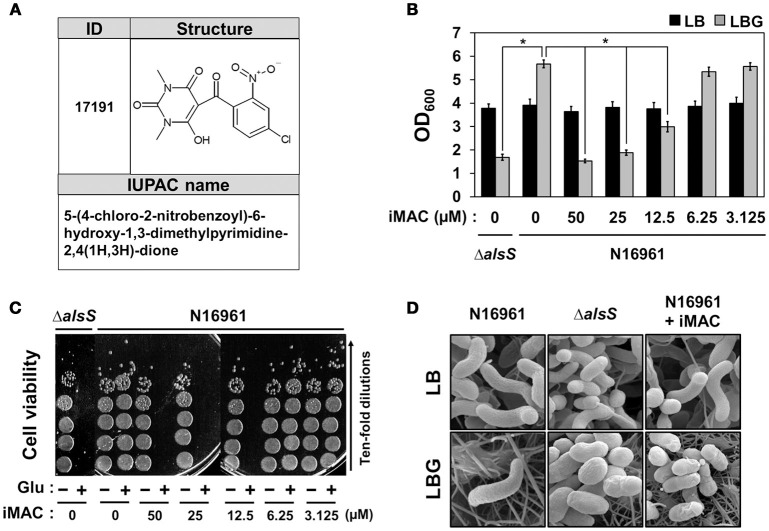
**N16961, the seventh pandemic El Tor strain, showed compromised viability in glucose-rich conditions in the presence of iMAC. (A)** The structure and IUPAC name of iMAC. **(B)** Bacterial cells were inoculated in LB or LBG with varying concentrations of iMAC and were grown for 16 h. Relative growth is calculated from OD_600_ measurements. ^*^p < 0.03 vs. OD_600_ values of LBG-grown cultures (iMAC untreated control). Data is presented as the mean ± *SD* (error bars) determined from three independent replicate experiments. **(C)** Changes in cell viability after treatment with iMAC. Aliquots of each culture were sampled at 16 h post-inoculation and were 10-fold serially diluted to assess the number of colony-forming units (CFUs). **(D)** Scanning electron microscope (SEM) images of *V. cholerae* strains grown in LB or LBG, in both the presence and absence of 50 μM iMAC treatment. Cells were grown for 16 h prior to processing for SEM. The images were acquired at 25,000x magnification.

We determined the chemical structure of iMAC, 5-(4-chloro-2-nitrobenzoyl)-6-hydroxy-1,3-dimethylpyrimidine-2,4(1H,3H)-dione, as shown in Figure 2A. In order to further investigate its effects on bacterial growth, we synthesized iMAC in a large quantity for all subsequent experimental use. We found that N16961 growth in LBG was significantly reduced by iMAC in a dose-dependent manner (Figure 2B), and resulting OD_600_ values of N16961 cultures decreased as the iMAC concentration increased. At iMAC concentrations <6.25 μM, we observed no growth inhibition. Importantly, the iMAC-induced growth defect was not observed in LB media, suggesting that iMAC is only effective under glucose-enriched conditions. Of note, the final OD_600_values of N16961 grown with 50 or 25 μM iMAC were similar to that of LBG-grown Δ*alsS* mutant, a non-acetoin-producing strain that was used as a negative control (Figure 2B). Moreover, the measured decrease in OD_600_ values was concomitant with the loss of viability (Figure 2C). In LBG, N16961 cells were completely killed after being treated with iMAC at concentrations of 12.5 μM or greater.

Our results strongly suggest that glucose-dependent iMAC-induced killing of N16961 occurs via the same mechanism that causes the Δ*alsS* strain to lose viability under the same conditions. In order to confirm this conclusion, we examined changes in cell shape using SEM (as described in Materials and Methods). As shown in Figure 2D, a typical curve-shape morphology was observed in cells grown in LB. In contrast, we observed spherically-shaped cells with knobby surfaces in LBG-grown Δ*alsS* mutant cells or in N16961 cells grown in LBG + iMAC (Figure 2D), conditions that lead to cell inviability. Together, these results clearly demonstrate that iMAC renders wild-type N16961 cells inviable under glucose-rich conditions, despite the fact that abundant glucose typically enhances bacterial growth.

### iMAC treatment alters the mode of glucose fermentation in N16961 cells

**Figure 3:**
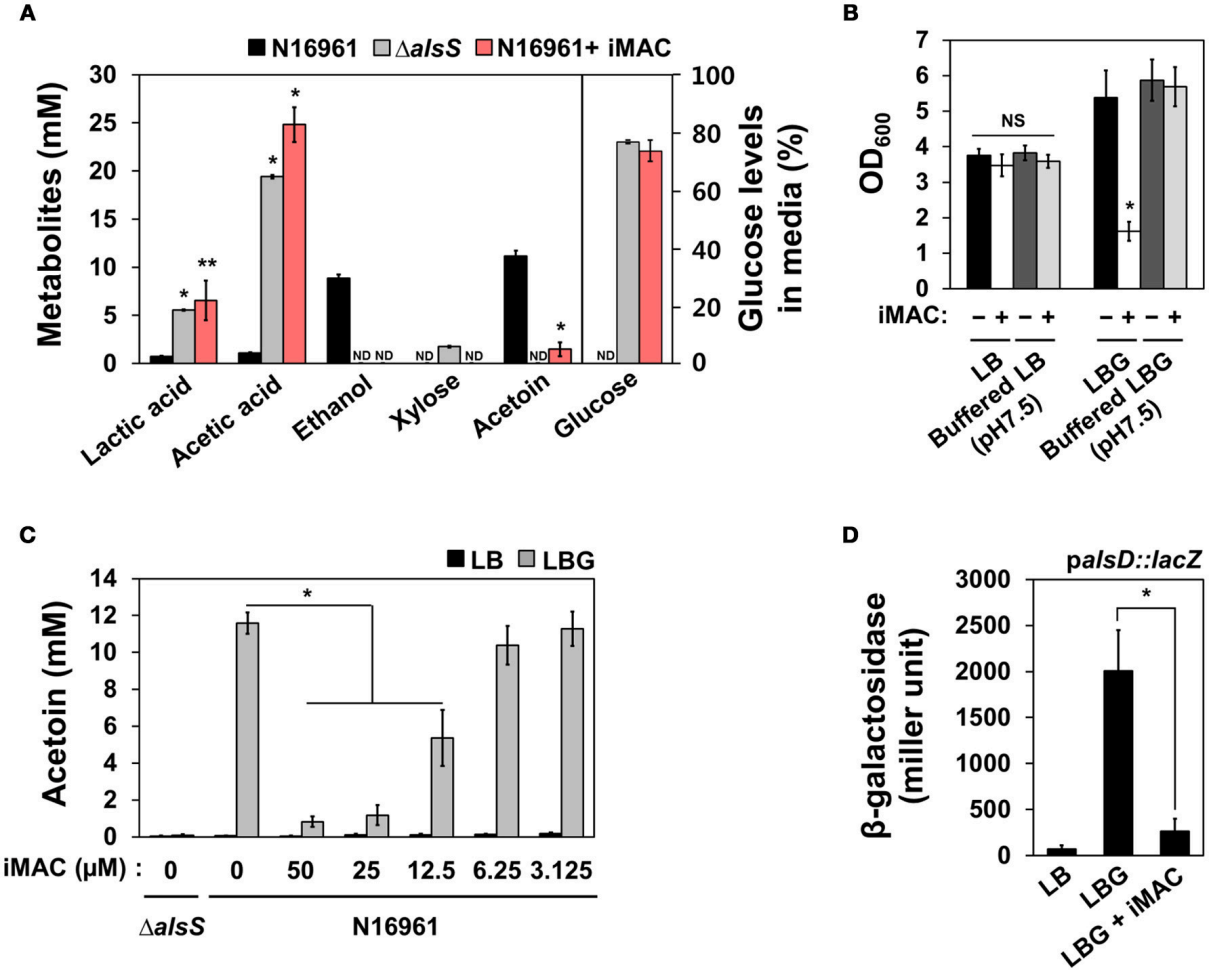
**iMAC modulates glucose metabolism and results in high levels of organic acids and low levels of acetoin, a neutral fermentation product. (A)** Metabolite and glucose levels in the culture supernatant were measured using HPLC as described in the Materials and Methods. ^*^p < 0.0001, ^**^p < 0.05 vs. metabolite levels that were measured in culture media of wild-type N16961 grown with LBG (iMAC untreated control). Non-detected values are displayed as “ND.” Data are presented as the mean ± *SD* (error bars) based on three independent replicate experiments. **(B)** Wild type N16961 strain was inoculated in phosphate-buffered (pH 7.5) or non-buffered LB/ LBG, respectively, either 50 μM iMAC treated or untreated, and were grown in aerobic conditions for 16 h. OD_600_ values were expressed as relative growth. ^*^p < 0.05 vs. OD_600_ value grown in buffered LBG with iMAC. Three independent experiments were performed, and values of mean ± *SD* (error bars) are displayed in each bar. **(C)** The acetoin level in the culture supernatant was measured by *Voges-Proskauer* (VP) tests for bacterial culture supernatants. Bacteria were cultured in each media for 16 h. ^*^p < 0.003 vs. acetoin levels in LBG grown cultures. **(D)** The promoter activity of the *alsD (VC1589)* gene in wild-type N16961 strain grown in LB, LBG, or LBG + 50 μM iMAC for 4 h. The reporter strain containing chromosomal *lacZ* reporter fusion was assayed in triplicate for β-galactosidase activity. Data are presented as the mean ± *SD*. ^*^p < 0.002 vs. β-galactosidase activity of cells grown in LBG.

To better characterize the manner in which glucose metabolism is influenced by iMAC treatment, we quantified an array of extracellular metabolites and measured the amount of glucose consumption in N16961 cells grown in LBG without or with iMAC. We performed the same set of measurements for the Δ*alsS* mutant strain as a control. Production of both lactic and acetic acids was significantly greater in N16961 when treated with iMAC (Figure 3A). The increased production of these two major organic acids is roughly equivalent to that of LBG-grown Δ*alsS* cells. This suggests that the iMAC-induced acidification was likely caused by accumulation of organic acids during glucose metabolism and suggests that mixed acid fermentation is stimulated by iMAC. When growth media were buffered with 50 mM phosphate to pH 7.5, the resultant drop in pH was not significant, and bacterial cells remained viable (Figure 3B). The production of ethanol and acetoin, two neutral fermentation products, was induced only in LBG-grown N16961, not in the control mutant strain (Figure 3A). Interestingly, N16961 produced non-detectable levels of ethanol and very little acetoin when treated with iMAC (Figure 3A, red bars). Similar to the Δ*alsS* mutant, iMAC-treated N16961 consumed only ~25% of the initial dose of glucose (Figure 3A) due to acidification and the resulting premature cell death. Glucose was completely consumed by iMAC-untreated N16961.

Acetoin production decreased as N16961 cells were treated with increasing concentrations of iMAC (Figure 3C) in a dose-dependent manner that is strikingly similar to that observed with OD_600_ values (shown in Figure 2B). The capability to produce acetoin, therefore, is critical for survival of N16961 under glucose-rich growth conditions. To better define the molecular mechanism by which iMAC modulates glucose metabolism, we measured the promoter activity of the acetoin biosynthesis operon in an N16961 reporter strain containing a p*alsD*::*lacZ* promoter fusion. N16961 cells were inoculated in LB, LBG, or LBG + 50 μM iMAC and grown for 5 h before harvest. Promoter activity was measured by β-galactosidase activity. The *alsD* promoter activity of cells grown in LBG was significantly greater than that of LB-grown cells (Figure 3D). However, the induction of the *alsD* promoter was significantly lower with iMAC treatment (Figure 3D). Together, these results suggest that iMAC acts upstream of transcriptional activation of acetoin biosynthesis gene.

### iMAC suppresses the production of the stringent response regulator (p)ppGpp in *V. cholera*

**Figure 4:**
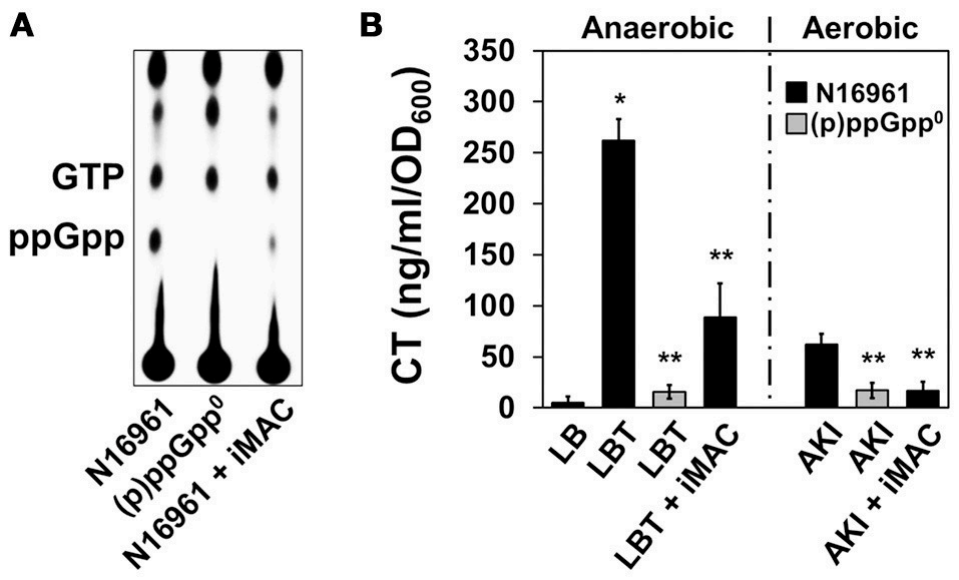
**Effects of iMAC on (p)ppGpp-dependent acetoin production. (A)** Intracellular (p)ppGpp levels were detected using TLC analysis. Wild-type N16961 and (p)ppGpp^0^ mutant (Δ*relA* Δ*spoT* Δ*relV* triple genes deletion mutant) were grown in LB or LB + 50 μM iMAC with P_32_-orthophosphate for 4 h. Cellular extracts were prepared as described in the Materials and Methods and analyzed using TLC. **(B)** CT levels in the culture supernatants were measured by CT-ELISA. Wild-type N16961 and (p)ppGpp^0^ mutant were grown under two different CT-inducible culture conditions as described in Materials and Methods. ^*^p < 0.003 vs. CT levels produced under grown in plain LB. ^**^p < 0.03 vs. CT levels of N16961 produced under grown in LBT. Data presented as the mean ± *SD* based on three independent replicate experiments.

Recent work from our group has demonstrated that the uninterrupted production of (p)ppGpp, a small nucleotide stress alarmone, is necessary to ensure balanced glucose metabolism in *V. cholerae* (Oh et al., 2015). Briefly, N16961 mutant cells that are incapable of producing (p)ppGpp also demonstrated inviability under glucose-rich conditions due to the acidification of their environment that results from the uncontrolled production of organic acids. Based on these results, we hypothesized that (p)ppGpp production is suppressed by iMAC treatment. To test this possibility, we compared the effect of (p)ppGpp deficiency with the effect of iMAC treatment in terms of growth, medium acidification, and cell viability. N16961 and its isogenic (p)ppGpp^0^ mutant (i.e., Δ*relA*Δ*relV*Δ*spoT* mutant) were inoculated in LB, LBG, or LBG + 50 μM iMAC and incubated for 16 h. We found that iMAC suppressed N16961 growth and acidified N16961 cultures in the presence of glucose to the same extent as the strain lacking (p)ppGpp production (Figures S3A,B). Likewise, the (p)ppGpp^0^ mutant also experienced environmental acidification in glucose-rich conditions, comparable to the levels measured in N16961 cells upon iMAC treatment (Figure S3C). Together, these results strongly suggest that the molecular mechanism by which iMAC affects *V. cholerae* is associated with the (p)ppGpp-dependent regulation of glucose metabolism. In order to confirm this, we measured the intracellular accumulation of (p)ppGpp with and without iMAC treatment. As shown in Figure 4A, a P_32_-labeled spot corresponding to ppGpp was substantially decreased in iMAC-treated N16961. The same spot was not detected in the (p)ppGpp^0^ mutant, confirming its lack of ppGpp production.

Prior work from our group has also demonstrated that cholera toxin (CT) production is significantly induced during anaerobic respiration after treatment with trimethylamine N-oxide (TMAO), which provides an alternative electron acceptor (Lee et al., 2012). Further, this increased production was suppressed when *V. cholerae* was unable to produce (p)ppGpp (Oh et al., 2014). In addition, CT production during the AKI condition (a biphasic growth to promote CT production; Iwanaga et al., 1986) is dependent on the cell's capability to produce (p)ppGpp (Oh et al., 2014). To investigate whether inhibition of (p)ppGpp production by iMAC treatment affects CT production, we measured the degree to which CT production is altered under each of these two conditions that normally stimulate CT production. We found that CT production was significantly decreased after iMAC treatment during anaerobic TMAO respiration (Figure 4B), although not as significantly was observed in the (p)ppGpp^0^ mutant. Additionally, iMAC treatment caused decreased CT production under the AKI condition (Figure 4B). Together, these results indicate that iMAC treatment causes *V. cholerae* wild-type cells to produce significantly less (p)ppGpp, an important regulator of the bacterial stringent response and virulence.

### Treatment with iMAC attenuated the colonization of *V. cholerae* in glucose-enriched *in vivo* conditions

**Figure 5:**
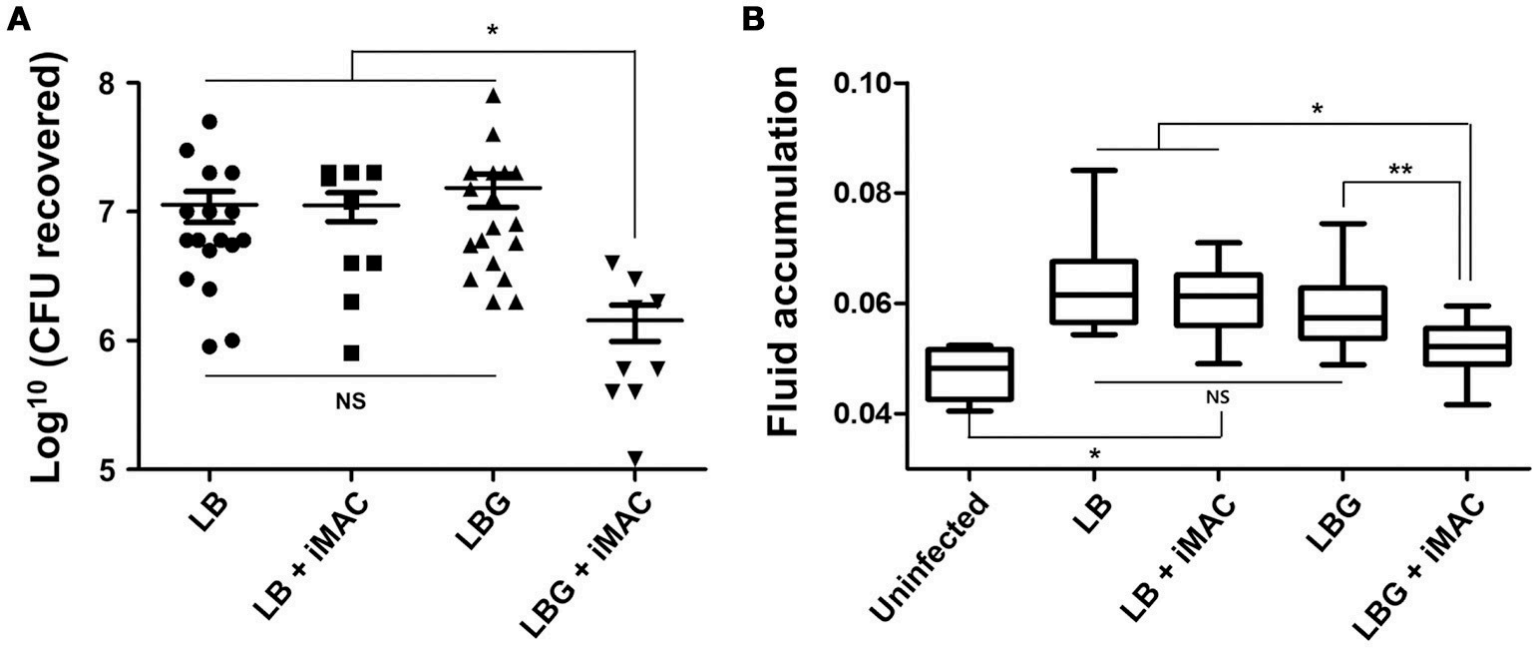
**Effects of iMAC on *V. cholerae in vivo* infectivity**. Infant mice (n > 8) were infected with 50 μL of each *V. cholerae* N16961 strain (1 × 10^6^ cells) suspended in indicated media supplemented with or without 50 μM iMAC. After 24 h, mice were sacrificed, and the entire intestines were extracted. **(A)** The number of viable cells was measured after 16 h by counting the number of viable bacterial cells recovered from the mouse intestine. The solid horizontal lines represent the geometric mean of each sample. ^*^p < 0.001 vs. CFU from infection with LB, LB + iMAC, or LBG. Each symbol shows the value obtained from each mouse. **(B)** The fluid accumulation ratio was calculated as described in Materials and Methods. Data are presented as the mean ± *SD* based on three independent replicate experiments. ^*^p < 0.001 vs. fluid accumulation ratio from uninfected control, ^**^p < 0.004 vs. fluid accumulation ratio from infection with LB, LB+iMAC, or LBG.

Next, we sought to characterize the effect of iMAC treatment on *V. cholerae* colonization and virulence *in vivo* by utilizing an infant mouse infection model. Six-day-old neonatal mice were infected with N16961 cells resuspended in LB or LBG media, both with and without the addition of 50 μM iMAC. When viable cells recovered from mouse intestine were enumerated, we found a significant decrease in CFU in mice infected with cells from media containing both glucose and iMAC (Figure 5A). No meaningful differences, however, were observed among the LB, LB + iMAC, and LBG groups (Figure 5A). Fluid accumulation (FA), a phenotype primarily caused by the presence of CT during infection, was also decreased in mice infected with bacterial cells prepared in LBG + iMAC (Figure 5B). Again, iMAC in LB exerted no effect on FA, which is consistent with previous findings that iMAC-mediated effects occur only in glucose-rich environments. This result is also consistent with our findings that iMAC suppresses CT production in glucose-rich environments (Figure 4B).

We next sought to assess whether iMAC treatment demonstrates any negative side-effects on host tissue cells. We cultured human intestinal epithelial cells (HT29) that were treated with iMAC and found no significant damage. Even when treated with increasing iMAC concentrations, no significant damage occurred (Figure S4A). Morphological change, typically an indication of cytotoxicity, was observed in HT29 cells treated with silver nanoparticles, which were used as a positive control agent (Figure S4A). After 48 h of treatment, MTT assays were performed to further confirm the lack of compromised epithelial cell viability after iMAC treatment. Whereas silver nano-particles exerted a clear cytotoxic effect on HT29 cells, iMAC treatment even at high concentrations failed to elicit any cytotoxic effect (Figure S4B).

### Several *V. cholerae* strains with different serotypes show similar effects after iMAC treatment

Evidence suggests that cholera pandemics and outbreaks are caused by *V. cholerae* strains of O1 or O139 serogroups. Other serogroups are sometimes associated with sporadic cholera-like diarrhea or localized outbreaks. The seventh cholera pandemic was caused by O1 serotype El Tor biotype strains, which gradually replaced the O1 classical biotype strains (Kim et al., 2015). The O139 serotype strains have also caused major endemic cholera (Barrett and Blake, 1981; Banerjee et al., 2014). Furthermore, O5 and O37 serogroups (referred to as non-O1/non-O139) have caused devastating cholera outbreaks in Czechoslovakia and Sudan, respectively (Aldová et al., 1968; Yamaichi et al., 1999; Rahaman et al., 2015). Given the diverse serotypes that show important clinical relevance, we investigated whether iMAC treatment causes similar glucose-dependent effects on viability in diverse *V. cholerae* strains. For this, we tested 22 different *V. cholerae* strains that were originally isolated either from patient samples or from the environment. We also included two related *Vibrio* species, as well as *Pseudomonas aeruginosa* and *Escherichia coli*. All strains were grown in LB or LBG medium in the presence or absence of 50 μM iMAC. After 16 h of growth, the pH of the media was measured, and the viability of the cells was determined, as summarized in Table 1. The O1 classical biotype strains showed a sharp decrease in viability in LBG media even without iMAC treatment, which is consistent with a previous report (Yoon and Mekalanos, 2006). Enhanced growth, however, was observed in all other *V. cholerae* strains, indicating that they can effectively metabolize glucose, leading to enhanced growth (Table 1). Importantly, we observed significant acidification of the media and decreased viability when all 21 of these robust “glucose metabolizers” were treated with iMAC. Other related *Vibrio* strains, including *V. vulnificus* and *V. paraheamolyticus*, showed no significant effects as a result of iMAC treatment, and they retained high viability in glucose-rich environments irrespective of the presence of iMAC (Table 1). These results strongly suggest that the effects of iMAC on glucose-dependent viability are specific and highly efficient in targeting *V. cholerae* strains, with no evident effects on other species or on host tissue cells.

**Table 1:** **Impact of iMAC on various *V. cholerae* strains and other related bacterial species**.

**Bacterial strain**		**Strain**	pH of culture media^*^				Cell viability^**^			
			**LB**	**LB + iMAC**	**LBG**	**LBG + iMAC**	**LB**	**LB + iMAC**	**LBG**	**LBG + iMAC**
*V. cholera*	O1, Classical	GP8	8.58 (±0.58)	8.69 (±0.03)	**4.73 (**±**0.36)**	**4.98 (**±**0.09)**	V	V	**NV**	**NV**
		A68	8.65 (±0.03)	8.69 (±0.01)	**4.41(**±**0.01)**	**4.89 (**±**0.02)**	V	V	**NV**	**NV**
		0395	9.12 (±0.18)	9.00 (±0.33)	**4.90 (**±**0.02)**	**5.02 (**±**0.17)**	V	V	**NV**	**NV**
		5698	7.62 (±0.21)	7.76 (±0.26)	**4.90 (**±**0.01)**	**5.13 (**±**0.05)**	V	V	**NV**	**NV**
		A76	8.73 (±0.04)	8.78 (±0.06)	**4.72 (±0.02)**	**5.10 (±0.01)**	V	V	**NV**	**NV**
	O1, El Tor	N16961	8.72 (±0.01)	8.74 (±0.02)	8.56 (±0.01)	**4.95 (**±**0.03)**	V	V	V	**NV**
		A10	8.79 (±0.03)	8.82 (±0.02)	8.47 (±0.06)	**4.99 (**±**0.01)**	V	V	V	**NV**
		A18	8.74 (±0.06)	8.80 (±0.01)	8.25 (±0.09)	**4.85 (**±**0.05)**	V	V	V	**NV**
		A152	8.85 (±0.05)	8.81 (±0.05)	8.48 (±0.01)	**4.97 (**±**0.08)**	V	V	V	**NV**
		A22	8.79 (±0.03)	8.83 (±0.02)	8.57 (±0.10)	**4.91 (±0.12)**	V	V	V	**NV**
	O1, South America	A200	8.72 (±0.03)	8.73 (±0.01)	8.60 (±0.03)	**4.87 (**±**0.11)**	V	V	V	**NV**
		A177	8.79 (±0.03)	8.81 (±0.03)	8.48 (±0.10)	**5.00 (±0.09)**	V	V	V	**NV**
	O1, US-Gulf	A213	8.62 (±0.01)	8.71 (±0.03)	8.51 (±0.03)	**4.86 (**±**0.05)**	V	V	V	**NV**
		A217	8.74 (±0.02)	8.80 (±0.02)	8.88 (±0.41)	**5.08 (±0.10)**	V	V	V	**NV**
	O139	M010	9.15 (±0.14)	9.19 (±0.10)	8.83 (±0.03)	**5.06 (**±**0.03)**	V	V	V	**NV**
		AR-196157	8.73 (±0.10)	8.72 (±0.01)	7.81 (±0.05)	**5.08 (**±**0.06)**	V	V	V	**NV**
		2206252	8.77 (±0.02)	8.77 (±0.05)	7.51 (±0.10)	**5.45 (±0.66)**	V	V	V	**NV**
	non-O1, non-O139	12/E-1776	8.78 (±0.12)	8.76 (±0.04)	8.58 (±0.01)	**4.91 (**±**0.02)**	V	V	V	**NV**
		14/E-1777	8.68 (±0.04)	8.63 (±0.22)	8.42 (±0.04)	**4.81 (**±**0.03)**	V	V	V	**NV**
		15/E-1877	8.82 (±0.04)	8.79 (±0.11)	8.64 (±0.21)	**4.82 (**±**0.21)**	V	V	V	**NV**
		A325	8.72(±0.01)	8.73 (±0.01)	8.55 (±0.11)	**4.87 (**±**0.04)**	V	V	V	**NV**
		A215	8.68 (±0 03)	8.80 (±0.01)	8.55 (±0.04)	**4.91 (**±**0.06)**	V	V	V	**NV**
*V. vulnificus*		M06-24/0	9.01 (±0.10)	9.08 (±0.09)	8.96 (±0.08)	9.00 (±0.11)	V	V	V	V
*V. paraheamolyticus*		ATCC 27519	9.07 (±0.08)	9.04 (±0.01)	9.01 (±0.02)	9.02 (±0.03)	V	V	V	V
*P. aeruginosa*		PA01	9.13 (±003)	9.20 (±0.05	8.70 (±0.12)	8.62 (±0.05)	V	V	V	V
*E. coli*		DH5a	8.66 (±0.19)	8.70 (±0.21)	**4.72 (**±**0.09)**	**4.71 (**±**0.13)**	V	V	V	V

* *pH values <5.5 are shown in bold*.

** *V, viable/NV, non-viable*.

## Discussion

Antibiotic-resistant strains of *V. cholerae* have been reported in the last decade; as such, it is vitally important to identify new concept antibiotics and/or develop novel therapeutics that can replace or otherwise supplement conventional cholera treatment (Kitaoka et al., 2011; Gupta et al., 2016). Previous work from the Mekalanos group has shown that a small-molecule inhibitor of *V. cholerae* virulence (known as virstatin; Hung et al., 2005) successfully represses CT and TCP production by inhibiting the dimerization of N-terminal domain of ToxT, a major virulence transcriptional activator (Shakhnovich et al., 2007a; Childers et al., 2011). Because virstatin specifically targets virulence, it has clinical use against many toxigenic *V. cholerae* strains that have emerged from O1/O139 serotypes. Virstatin resistance, however, has recently emerged in *V. cholerae* strains derived from non-O1/non-O139 serogroups that have recently caused sporadic outbreaks (Shakhnovich et al., 2007b). This highlights the importance of identifying new antibiotics that target *V. cholerae* cells via a common molecular mechanism. While *V. cholerae* is quite tolerant to alkaline conditions, it is highly sensitive to even mildly acidic conditions, a potential vulnerability that could be exploited in clinical treatments (Finkelstein, 1996). Because iMAC targets this physiological characteristic that is a conserved mechanism of *V. cholerae*, it exhibits broad applicability, suggesting a more effective common antibiotic against cholera.

We successfully identified iMAC as a result of the following contributions. First, we used a detection method of medium acidification that used a pH indicator and glass beads, the latter of which effectively homogenized the media with gentle shaking and therefore reflected an accurate pH measurement, which also enabled us to screen in a high-throughput format. Second, it is known that glucose metabolism in *V. cholerae* bifurcates into two major pathways, one that produces organic acids and another that produces neutral end-products including acetoin and ethanol. Upon activation, acetoin production suppresses the production of organic acids, and vice versa. This suggests that modulating glucose metabolism toward the production of organic acids can provide a novel strategy for the targeted killing of *V. cholerae* cells. This potential is further bolstered by the fact that *V. cholerae* is highly sensitive to acid stress in its environment (Merrell and Camilli, 2000). Based on our results in Table 1, the *E. coli* strain DH5α maintained its viability down to a pH of ~4.7, while all *V. cholerae* strains were inviable at pH around 5.0. Finally, a *V. cholerae* mutant that cannot produce (p)ppGpp also becomes inviable in glucose-rich conditions (Oh et al., 2015). This previous finding suggests that (i) any chemical that interferes with (p)ppGpp production or antagonizes its action can cause inviability under glucose-rich environments and, (ii) therefore, *V. cholerae* glucose metabolism provides an excellent target for chemical treatment.

One of the long-standing questions in cholera epidemiology is how the El Tor biotype strains came to replace classical biotype strains (Pradhan et al., 2013; Kim et al., 2014). Our work shows that the classical biotype strains cannot conduct acetoin production during glucose fermentation even in LBG medium (Table 1), a phenotype identical to that of the N16961 (p)ppGpp^0^ mutant. This suggests that the classical biotype strains might have been less competent than the El Tor biotype in producing (p)ppGpp under glucose-rich conditions, such as those commonly found in the human intestinal environment. This also suggests that El Tor biotype strains have evolved to more efficiently mount the stringent response (SR), which would further increase their fitness in the hostile host intestine relative to the classical biotype strains. Of note, classical biotypes have commonly been considered to be more virulent than the El Tor strains. The production of CT and TCP by the classical strains was significantly greater *in vivo* than in the El Tor strains (Turnbull et al., 1985; Jonson et al., 1992). In addition, the classical strains exhibit higher expression of virulence-associated genes (Beyhan et al., 2006). Petritsch and colleagues have also reported that diarrheal volumes increase when higher levels of CT are found inside the human intestine (Petritsch et al., 1992). Rapid bacterial expulsions, therefore, might have occurred more frequently during infection by classical biotype strains compared to the El Tor strains. Along these lines, it is thought that El Tor biotype strains were better adapted to the host intestine by having lower virulence expression and increased capability to activate SR, allowing the El Tor strains to outcompete the classical strains in nature.

The stringent response (SR), which is activated under conditions of nutrient starvation, also plays an important role in regulating bacterial virulence (Dalebroux et al., 2010). Previous work from our group has demonstrated that, in *V. cholerae*, (p)ppGpp is required for glucose metabolism, and its production is essential for bacterial growth (Oh et al., 2015) and CT production (Oh et al., 2014). These findings raise the possibility that: (i) (p)ppGpp regulates a common mechanism that controls both bacterial metabolism and virulence (two features that otherwise appear to be distinctly regulated); and (ii) that intervening and blocking this mechanism provides a promising alternative to treat *V. cholerae* infection. Recently, Wexselblatt and colleagues showed that a chemical compound (which they termed “relacin”) suppresses the production of (p)ppGpp in Gram-positive bacterial species (Wexselblatt et al., 2012). Relacin is an analog of (p)ppGpp, and it was found to inhibit RelA, a conserved enzyme responsible for (p)ppGpp synthesis. However, relacin is required in significant quantities (~mM levels) in order to exert an inhibitory effect on bacterial growth and/or viability. Our results indicate that a relatively low concentration of iMAC (12.5 μM) successfully suppresses (p)ppGpp production and other downstream events. Although more experiments are necessary to precisely define the molecular mechanisms by which iMAC represses (p)ppGpp production, it is nonetheless clear that iMAC-mediated regulation occurs much more efficiently than that by relacin.

We also tested the effect of iMAC on several El Tor strains, including four prototype El Tor strains collected in south Asia and Mozambique, two strains collected during cholera epidemics in Latin America in 1990s, and two U.S. Gulf-Coast strains that are considered to be pre-seventh pandemic strain (Mutreja et al., 2011). Three O139 serogroup strains, which are considered to be a subgroup within El Tor biotype strains, were also tested. Medium acidification and reduced viability after treatment with iMAC in glucose-rich conditions were common phenomena for all of the tested *V. cholerae* strains, demonstrating that iMAC exerts its effect generally across the El Tor strains.

**Figure 6:**
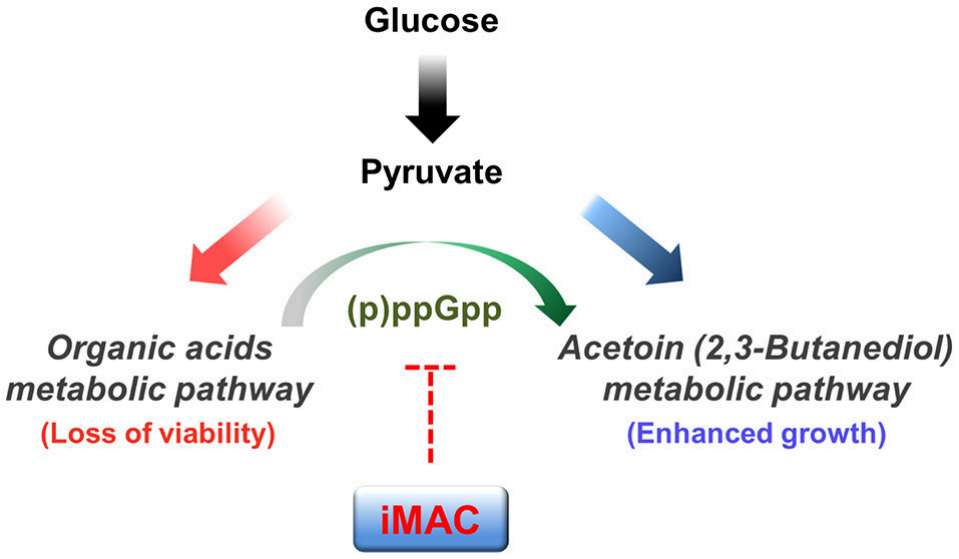
**A potential mechanism by which iMAC, acting as a ppGpp-dependent metabolic switch, induces inviability of *V. cholerae***. In the presence of glucose, *V. cholerae* normally metabolize glucose by the mixed acid fermentation during early stages. As growth proceeds, the environmental pH decreases as a result of the accumulation of organic acid metabolites. The glucose metabolic pathway then switches to acetoin fermentation to avoid lethal acidification. Previous work demonstrates that intracellular (p)ppGpp has a critical role in this glucose metabolic switch. Here, iMAC is characterized in terms of its potential role in inhibiting the accumulation of intracellular (p)ppGpp. Overall, iMAC successfully prevents the ppGpp-dependent glucose metabolic switch by inhibiting the accumulation of intracellular ppGpp. As a result, cells lose their viability as their environment becomes acidified as a result of uncontrolled organic acid fermentation in glucose-enriched conditions.

In conclusion, we identified a novel compound that specifically kills *V. cholerae* in glucose-rich environments via a mechanism that suppresses (p)ppGpp production. We propose that iMAC acts as a metabolic switch to inhibit acetoin fermentation (as summarized in Figure 6). We also show that iMAC elicits no clear toxicity to human cells or other more distantly related bacteria. These findings suggest that iMAC could be a highly effective tool for treating *V. cholerae* infections. In particular, we suggest that iMAC could potentially act synergistically with oral rehydration solution (ORS) therapy, because the continuous administration of ORS creates a glucose-rich environment in the host intestine within a short time. We expect these results to help establish better strategies for treating cholera patients.

## Author contributions

YO and SY conceived, designed and coordinated the study. YO, HYK, EK, JG, WH, and DK performed the experiment and acquired the data. HRK provided the reagent for the study. SY, YO, and DK wrote the manuscript. All the authors participated in discussions of the results and reviewed the final draft.

### Conflict of interest statement

The authors declare that the research was conducted in the absence of any commercial or financial relationships that could be construed as a potential conflict of interest.
