# Supplementary material for: Selective and Efficient Elimination of Vibrio cholerae with a Chemical Modulator that Targets Glucose Metabolism
Source: Front Cell Infect Microbiol. 2016 Nov 16;6:156. doi: 10.3389/fcimb.2016.00156 (PMC5111416; doi:10.3389/fcimb.2016.00156)
Supplement: Supplementary file 1 [file DataSheet1.PDF]

[Supplementary Information]

Selective and Efficient Elimination of *Vibrio cholerae* with a Chemical Modulator that Targets Glucose Metabolism

Young Taek Oh, Hwa Young Kim, Eun Jin Kim, Junhyeok Go, Wontae Hwang, Hyoung Rae Kim, Dong Wook Kim, and Sang Sun Yoon

☐ **Contents**

- Table S1
- Figure S1
- Figure S2
- Figure S3
- Figure S4
- References

Table S1. Bacterial strains used in this study

Strains	Relevant characteristics	Reference
Vibrio cholerae		
N16961	Wild type, O1 serogroup, *El Tor* biotype	[1]
ΔalsS	N16961, *VC1590* (*alsS*) gene deleted	[2]
p*VC1589*::*lacZ*	N16961, *VC1589* promoter *lacZ* reporter fusion	[2]
(p)ppGpp°	N16961, *relA*, *relV* and *spoT* genes deleted	[3]
GP8	O1 serogroup, Classical biotype (India)	[4]
A68	O1 serogroup, Classical biotype (Egypt)	[4]
O395	O1 serogroup, Classical biotype (India)	[4]
569B	O1 serogroup, Classical biotype	[5]
A76	O1 serogroup, Classical biotype (Bangladesh)	[4]
A10	O1 serogroup, El Tor biotype (Bangladesh)	[4]
A18	O1 serogroup, El Tor biotype (India)	[4]
A152	O1 serogroup, El Tor biotype (Mozambique)	[4]
A22	O1 serogroup, El Tor biotype (Bangladesh)	[4]
A200	O1 serogroup, South America (Argentina)	[4]
A177	O1 serogroup, South America (Colombia)	[4]
A213	O1 serogroup, US-Gulf (Georgia)	[4]
A217	O1 serogroup, US-Gulf (Louisiana)	[4]
MO10	O139 serogroup (India)	[4]
AR-196157	O139 serogroup (Mozambique)	[6]
2206252	O139 serogroup (Mozambique)	[6]
12/E-1776	Non-O1 serogroup	[6]
14/E-1777	Non-O1 serogroup	[6]
15/E-1877	Non-O1 serogroup	[6]
A325	Non-O1 serogroup (Argentina)	[4]
A215	Non-O1 serogroup (California)	[4]
Vibrio vulnificus		
MO6-24/O	Clinical isolate	[7]
Vibrio parahaemolyticus		
ATCC27519		[8]
Pseudomonas aeruginosa		
PAO1	Wild type	[9]
Escherichia coli		
DH5α	(Φ80 *lacZ ΔM15*) *recA1 endA1 gyrA96 relA1 thi-1 hsdR17* (r_K^- m_K^-) *supE44 deoR* (*lacZYA-argF*) *U169*	[10]

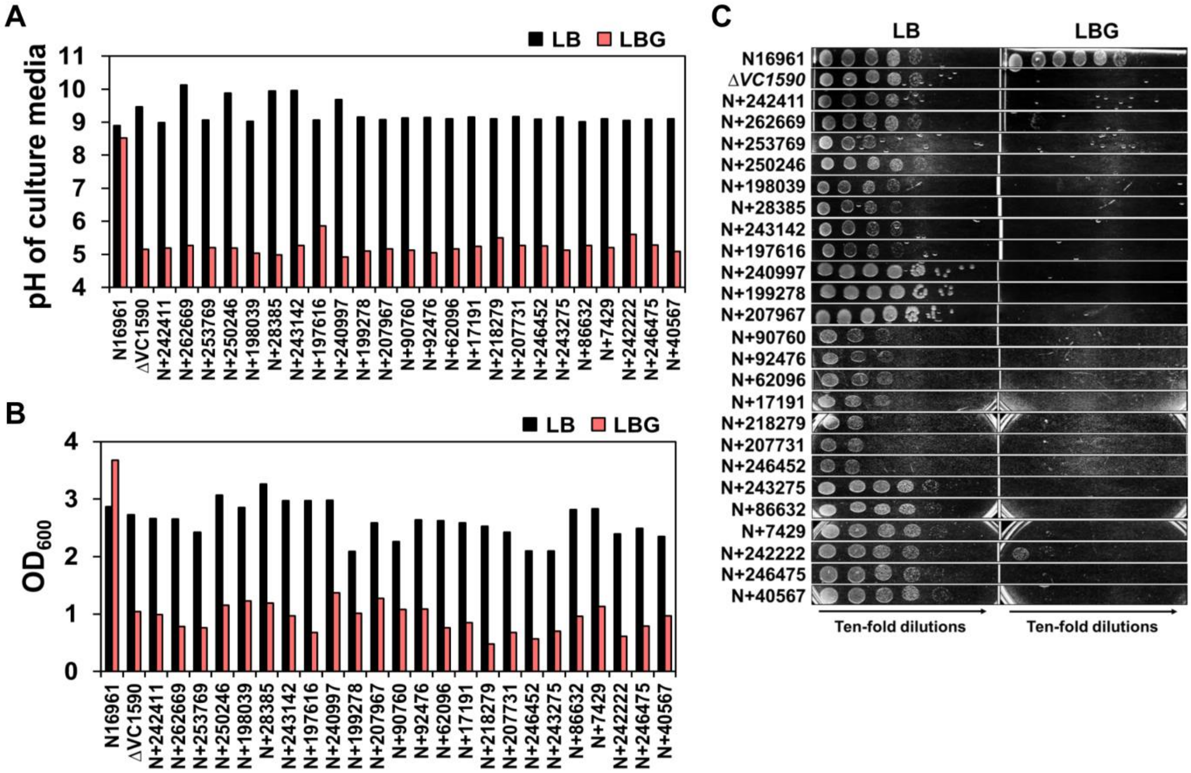

Figure S1. Characterization of compounds selected from primary screening. The wild-type N16961 strain and the *VC1590* (*alsS*) deletion mutant strain were inoculated in LB or LBG (LB containing 1% glucose) with 50 μM of each tested compound and were grown in aerobic conditions for 16 hrs in a 15 mL test tube. (A) Twenty-four compounds demonstrated the capacity to inhibit growth in LBG media. OD_{600} was measured, and growth rates are presented as relative growth. (B) Media acidification was observed in bacterial cultures grown with each of the 24 compounds treated in LBG medium. The media pH was measured in aliquots of culture supernatants. (C) iMAC induces loss of viability during overnight growth in LBG medium. Bacterial cell viability is represented in terms of number of colony-forming units (CFU).

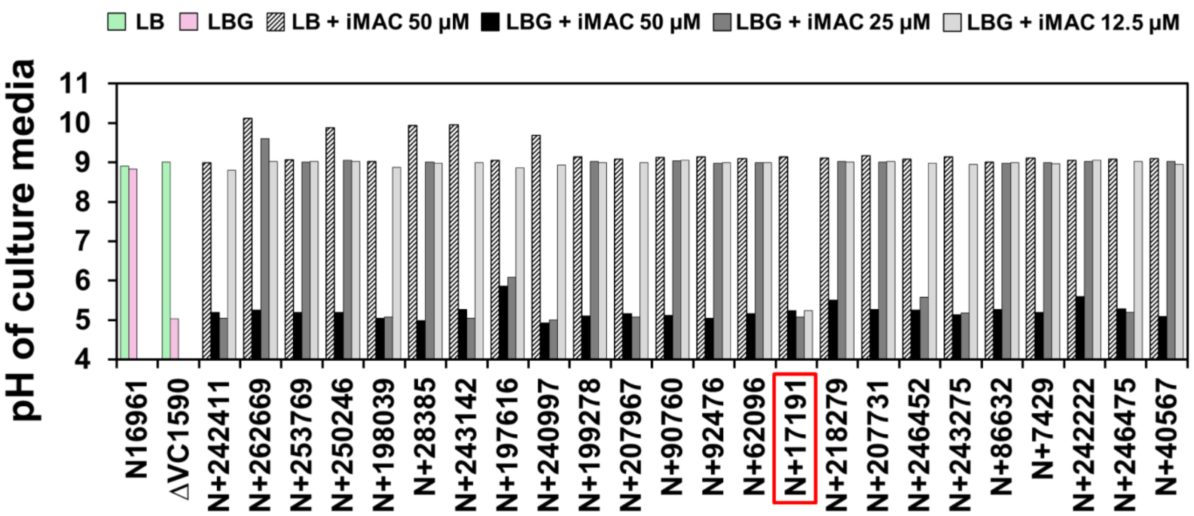

Figure S2. Selection of iMAC17191, which exhibits activity at the lowest concentration. To identify the most effective compound, two-fold serial dilutions of each compound were added to LB or LBG media, and cells were grown for 16 hrs. The degree of media acidification was quantified by comparing the pH value of each compound to the pH value of the untreated control. Compound No.17161 was the most effective at inducing a decrease the pH of the media (highlighted in the red box).

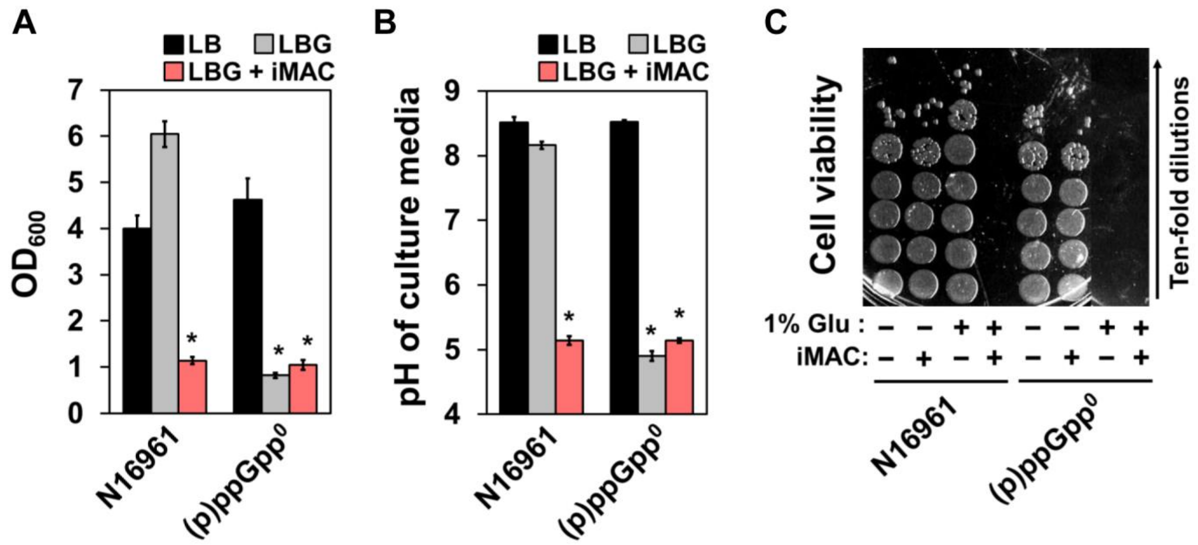

Figure S3. Effects of iMAC on (p)ppGpp-dependent acetoin production. Wild-type N16961 and (p)ppGpp^0 mutant strains were inoculated in LB, LBG, or LBG + 50 µM iMAC and were grown for 16 hrs. (A) OD_{600} values were expressed as relative growth. **p*<0.003 vs. OD_{600} values of LB grown cultures. (B) Change in the pH of the media. Data are presented as the mean ± SD based on three independent replicate experiments. **p*<0.001 vs. medium pH of LB grown cultures. (C) Changes in bacterial cell viability.

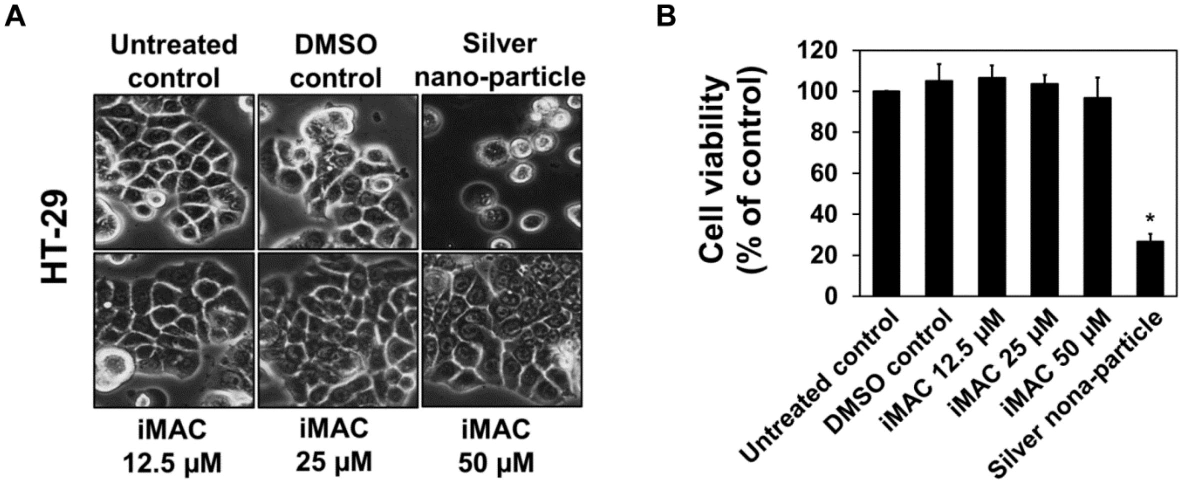

Figure S4. Cytotoxic effect of iMAC on human intestinal epithelial cells. (A) 1×10^6 cells/well of human intestinal epithelial cells (line HT-29) were cultured in a 6-well plate and exposed to the indicated concentrations of iMAC17191. Images of viable cells were obtained by optical microscopy after 48 h. Cells treated with silver nanoparticles served as a negative control. (B) To determine cell viability, the colorimetric MTT metabolic activity assay was used as described in the Methods section.

References

1. Fullner KJ, Mekalanos JJ. Genetic characterization of a new type IV-A pilus gene cluster found in both classical and El Tor biotypes of Vibrio cholerae. *Infect Immun* **67**, 1393-1404 (1999).
2. Oh YT, Lee KM, Bari W, Raskin DM, Yoon SS. (p)ppGpp, a Small Nucleotide Regulator, Directs the Metabolic Fate of Glucose in Vibrio cholerae. *The Journal of biological chemistry* **290**, 13178-13190 (2015).
3. He H, Cooper JN, Mishra A, Raskin DM. Stringent response regulation of biofilm formation in Vibrio cholerae. *Journal of bacteriology* **194**, 2962-2972 (2012).
4. Mutreja A, *et al.* Evidence for several waves of global transmission in the seventh cholera pandemic. *Nature* **477**, 462-465 (2011).
5. Yoon SS, Mekalanos JJ. 2,3-butanediol synthesis and the emergence of the Vibrio cholerae El Tor biotype. *Infection and immunity* **74**, 6547-6556 (2006).
6. Lee JH, *et al.* Multilocus sequence typing (MLST) analysis of Vibrio cholerae O1 El Tor isolates from Mozambique that harbour the classical CTX prophage. *Journal of medical microbiology* **55**, 165-170 (2006).
7. Kim MJ, Kim J, Lee HY, Noh HJ, Lee KH, Park SJ. Role of AcsR in expression of the acetyl-CoA synthetase gene in Vibrio vulnificus. *BMC microbiology* **15**, 86 (2015).
8. Noh HJ, *et al.* Role of VcrD1 protein in expression and secretion of flagellar components in Vibrio parahaemolyticus. *Archives of microbiology* **197**, 397-410 (2015).
9. Yoon MY, Lee KM, Park Y, Yoon SS. Contribution of cell elongation to the biofilm formation of Pseudomonas aeruginosa during anaerobic respiration. *PLoS One* **6**, e16105 (2011).
10. Taylor RG, Walker DC, McInnes RR. E. coli host strains significantly affect the quality of small scale plasmid DNA preparations used for sequencing. *Nucleic acids research* **21**, 1677-1678 (1993).
